# Editorial: Plant's bioactivity in modern health and diet: benefits, limitations and trendy application

**DOI:** 10.3389/fnut.2024.1385444

**Published:** 2024-03-11

**Authors:** Aleksandar Ž. Kostić, Dasha Mihaylova, Nadezhda Traycheva Petkova

**Affiliations:** ^1^Faculty of Agriculture, University of Belgrade, Belgrade, Serbia; ^2^Department of Biotechnology, University of Food Technologies, Plovdiv, Bulgaria; ^3^Department of Organic Chemistry and Inorganic Chemistry, University of Food Technologies, Plovdiv, Bulgaria

**Keywords:** biological active compounds, original research, systematic review, review, plant‘s bioactivity

Plant's Bioactivity in Modern Health and Diet: Benefits, Limitations, and Trendy Application is a Frontiers in Nutrition Research Topic that is dedicated to plants as an inexhaustible source of beneficial bioactive substances and aims to follow the increased interest in foods with added nutritional value.

We received seven submissions; three of these were declined following an initial editorial assessment or reviewers' comments. Four articles were accepted after one or more rounds of peer revision as follows: 1 review, 1 systematic review, and 2 original research documents, one of which was a clinical trial and published in the collection of articles under the Research Topic “*Plant's Bioactivity in Modern Health and Diet: Benefits, Limitations, and Trendy Application*.”

Cheng-yuan and Jian-gang presented a review article with hyperuricemia (HUA) as a topic. They summarized the constant search for potential medicinal and edible plants to prevent this common metabolic disease and their bioactives, in particular, as a future perspective. Five categories, flavonoids, phenolic acids, alkaloids, polysaccharides, and saponins, were mentioned with the potential to exhibit positive uric acid-lowering effects by inhibiting uric acid production, promoting uric acid excretion, and improving inflammation. *Camellia sinensis, Cichorium intybus* L., *Chaenomeles sinensis, Citrus limon, Sophora japonica* Linn, *Folium nelumbinis, Chrysanthemum* flowers, and some other medicinal and edible plants were mentioned as of potential interest in this regard. The authors summarized several bioactive substances and their uric acid-lowering mechanism and did not forget to mention potential pressing problems in treating HUA with medicinal plants. However, they pointed out that medicinal plants presented a potentially effective strategy for the prevention and treatment of HUA, mainly based on their active ingredients that provide new directions for the synthesis of effective and less toxic drugs for the treatment of HUA.

Mohan et al. conducted a clinical trial in an attempt to explore the short-term influence of a proprietary oil extract of black cumin (*Nigella sativa*) on non-restorative sleep. The study had a randomized, double-blinded, placebo-controlled actigraphy design. The clinical trial was conducted with healthy male and female participants (*n* = 70), aged 18–65 years (BMI 22–28 Kg/m^2^), who were randomized to either placebo or BCO-5 (*n* = 35/group). Significant improvement in participants with non-restorative sleep problems was found with 200 mg/day supplementation for 7 days, indicating the potential role of black cumin extract as a natural sleep aid.

Uehara et al. investigated the policosanol supplementation effect on high-density lipoprotein (HDL)-cholesterol (HDL-C), demonstrating that the policosanol supplementation increased the HDL cholesterol efflux capacity (CEC) in healthy participants. The authors conducted a random study with healthy Japanese participants aiming to establish the effect of policosanol supplementation regarding HDL function, HDL-related factors, and HDL morphology/component. The authors pointed out the limitations of this topic and the need for further investigations to establish the evidence of policosanol's effects in the clinical setting. The lipoprotein analysis was conducted on 32 healthy Japanese participants (placebo, *n* = 17, or policosanol supplementation for 12 weeks, *n* = 15) from a randomized Cuban policosanol clinical trial. However, the association between Cuban policosanol supplementation and HDL CEC, an important function of HDL, remains unclear.

Jafarpour et al. investigated sumac consumption with respect to its effect on cardiovascular parameters. The authors summarized several databases, including PubMed/Medline, SCOPUS, and ISI Web of Science until January 2023. The systematic review summarized the results of 16 randomized controlled trials. Sumac was reported to significantly affect low-density lipoprotein, high-density lipoprotein, triglycerides, fasting blood glucose, insulin, homeostasis model assessment of insulin resistance (HOMA-IR), and anthropometric indices. The results indicated a significantly reduced total cholesterol when the intervention duration was ≥12 weeks. Based on this, sumac consumption could be recommended to improve the overall cardiometabolic status of patients.

In conclusion, we are grateful to “Frontiers in Nutrition” for the opportunity to serve as editors of this Research Topic, which is of great interest to us. It was a motivating and inspiring experience from which we learned a lot, and we look forward to continuing our work in this area. We would also like to thank our valued authors for their contributions and for sharing their research in this collection, which we believe will be meaningful not only to them but also to the readers. Last but not least, we would like to thank the reviewers for their time and input, which undoubtedly improved the quality of the articles accepted for publication.

## Author contributions

AK: Writing—original draft, Writing—review & editing. DM: Writing—original draft, Writing—review & editing. NP: Writing—original draft, Writing—review & editing.

